# Supplementary material for: A selective and augmentable butyrate-FFAR2 signal circuitry programs the cellular identity of enteroendocrine L-cells
Source: Commun Biol. 2026 Mar 17;9:606. doi: 10.1038/s42003-026-09830-5 (PMC13144487; doi:10.1038/s42003-026-09830-5)
Supplement: Supplementary file 2 — Supplementary Information [file 42003_2026_9830_MOESM2_ESM.pdf]

Supplementary Material

A selective and augmentable butyrate-FFAR2 signal circuitry programs the cellular identity of enteroendocrine L-cells

Aanya Hirdaramani, Chia-Wei Cheng, Aylin C. Hanyaloglu, Gary Frost

Figure S1. Validation of nutrient-sensing machinery and hormone repertoire characteristic of L-cells in the human colonic NCI-H716 cell line, related to Figure 1

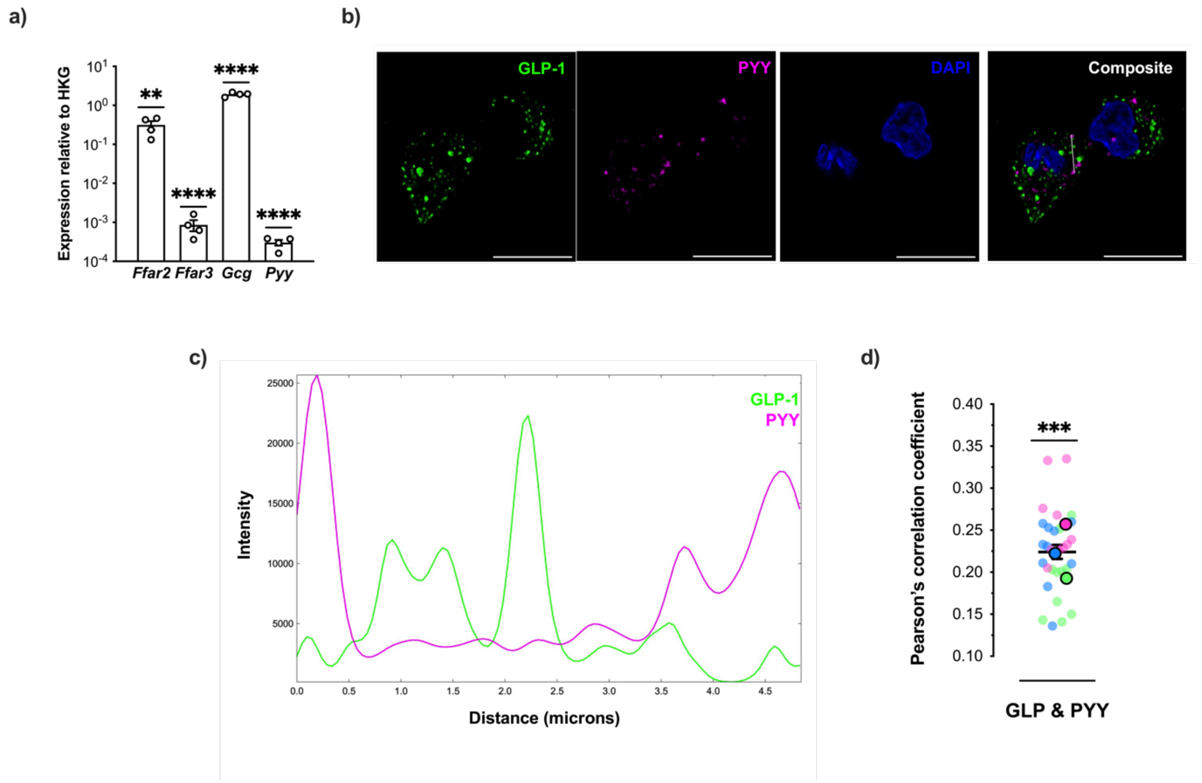

(a) *Ffar2, Ffar3, Gcg* and *Pyy* transcript expression in NCI-H716 cells were detected by RT-qPCR. Gene expression relative to ribosomal protein L12 (*Rpl12)* housekeeping gene (HKG) was determined using the $2^{-\Delta\Delta Ct}$ method with ribosomal protein L12 (*Rpl12)* as a housekeeping gene control. Data are plotted as the mean ± SEM of fold-change ($2^{-\Delta\Delta Ct}$) values. Symbols depict the mean ± SEM from n=3 biological repeats. Statistical significance was assessed on ΔCt values. (**p<0.01; ****p<0.0001, t-test vs HKG). **(b-d)** NCI-H716 cells were stained with anti-GLP-1 and anti-PYY antibodies captured by confocal microscopy in super-resolution by an adaptive deconvolution module (LIGHTNING, Leica). **(b)** Representative confocal immunofluorescent images of anti-GLP-1 and anti- PYY staining in NCI-H716 cells Scale bar= 10μM. Images are representative of 40 cells imaged across 6 independent experiments**.** **(c)** Line plot of GLP-1 and PYY intensity profiles across a ROI (indicated by a white line in (A) of 5μM length) **(d)** Pearson's correlation coefficient of GLP-1 and PYY channel overlap in confocal images. Data represented as mean ± SEM of n=30 cells collected across n=3 independent experiments, coloured by independent repeats. Values from individual cells are shown as smaller data points with colours corresponding to the respective experimental run. One sample t-test; Pearsons vs 1 (***p<0.001).

Figure S2 Butyrate-mediated Gαi signalling is inhibited by pertussis toxin and impact of FFAR2 antagonism, G-protein inhibitors, or inhibition of dynamin-mediated internalization on PYY secretion in NCI-H716 cells, related to Figure 3.

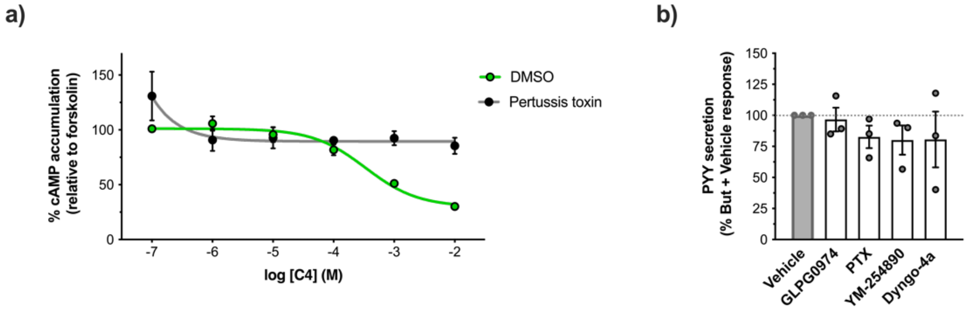

(A) Intracellular cAMP accumulation measured in NCI-H716 cells pre-treated with DMSO (vehicle) or Pertussis toxin (500ng/ml, 20 hours) and then stimulated with 3-isobutyl-1- methylxanthine (IBMX) for (500nM,5 min) followed by forskolin (FSK) (3uM) in the presence of increasing doses of But for 5 min. Data are expressed as % response of cAMP accumulation in FSK-treated cells and represent the mean ± SEM from n=3 independent experiments. **(B)** Secretion of PYY from NCI-H716 cells pre-treated with DMSO (Vehicle), GLPG0974 (1μM, 15 min), PTX (500ng/ml, 20 hours), Gαq inhibitor YM-254890 (10nM, 15 min) or dynamin-dependent endocytosis inhibitor Dyngo-4a (50uM, 45 min) followed by incubation with 2mM But for 24h. Cells were incubated with ligand-free secretion buffer for 2hr and supernatants were subsequently assayed for PYY concentration by ELISA. Data was normalised as fold change over untreated cells, and in (d), normalised But with Inhibitor response are shown as % (depicted by dotted line at 100%). Symbols depict the mean ± SEM from n=3 independent experiments.

Figure S3 Positive allosteric modulation of SCFA-mediated FFAR2-Gαi signalling by AZ-1729 in NCI-H716 cells, related to Figure 4.

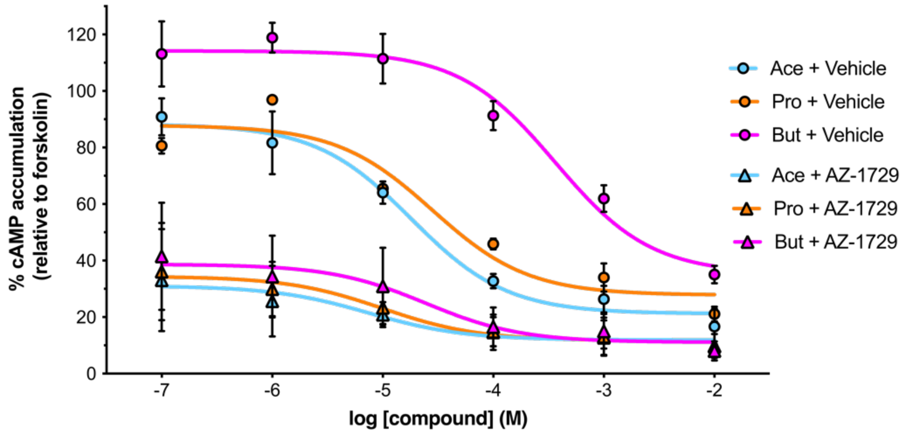

Positive allosteric agonism of SCFA- mediated Gαi responses in NCI-H716 cells by AZ-1729. Cells were treated with IBMX (500nM, 5 min), followed by forskolin (FSK) (3μM, 5 min) or combination of FSK (3μM, 5min) and NaCl/SCFA (1mM, 5 min) and DMSO vehicle, or a combination of FSK (3μM, 5min), and NaCl/SCFA (1mM, 5 min), and AZ-1729 (1μM, 5 min). Data represents mean ± SEM (for NaCl) or mean (for SCFAs) from n=3 independent experiments.

Figure S4 Validation of Hes1 expression in the Mouse large intestine, measurements of total organoid area and immunofluorescent staining for PYY^{+}cells in Hes1-GFP organoids, related to Figure 5

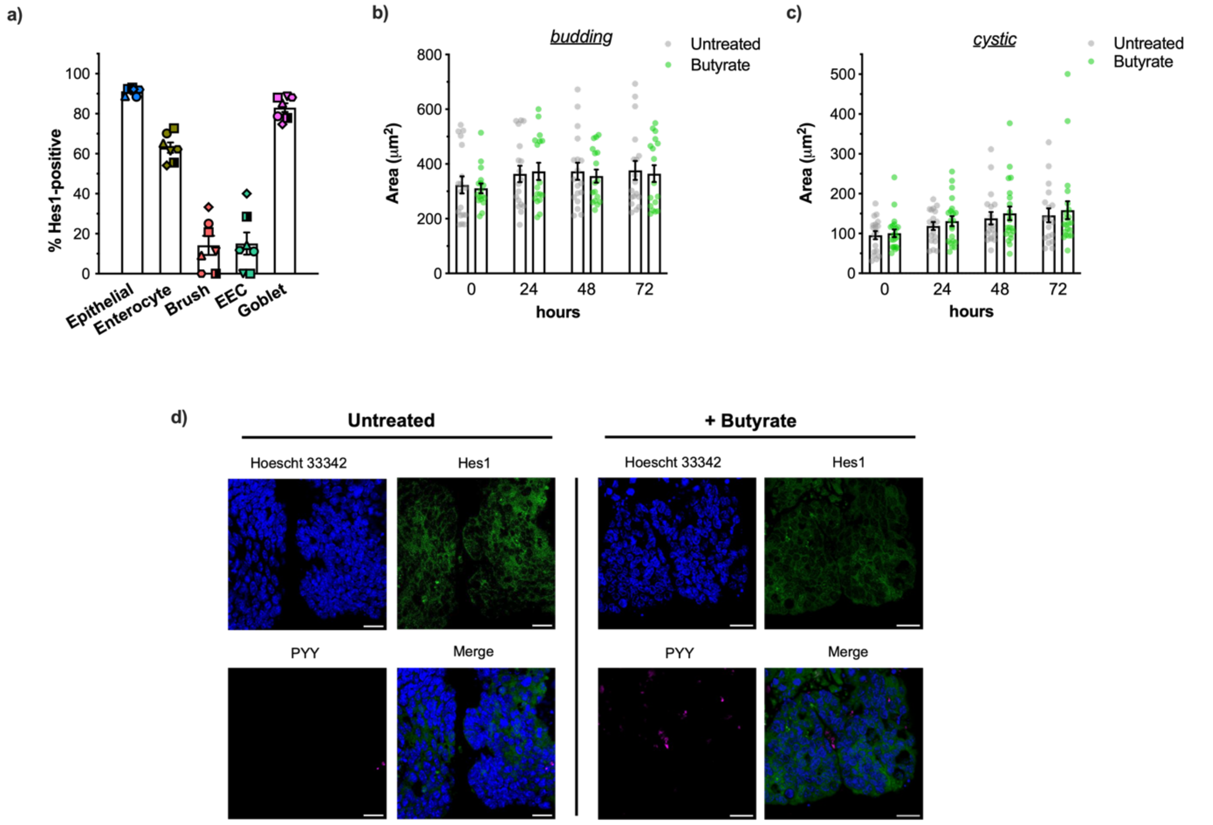

(a) Comparison of Hes1 expression across cellular ontologies in the large intestine, by analysis of the single-cell RNA-seq data from the Tabula Muris project. Data are shown as % of cells per ontology class with log_2CPM-normalised Hes1 expression > 0 in Tabula Muris. Distinct symbol shapes represent mean % per cell ontology class in individual mice, n=7 mice**.** Total area of (b) budding and (c) cystic Hes1-GFP organoids monitored across a 72 hour timeframe in untreated culture conditions (Control) or cultures incubated with 2mM of sodium butyrate (But) from 24 hours. Data are from n=18 'budding' organoids and n=19 'cystic' organoids, and were collected across cultures from three separate Hes1-GFP mice. (d) Representative confocal images of Hes1-GFP organoids in untreated culture conditions or cultures incubated with 2mM butyrate for 48h, stained for anti-PYY antibody and Hoescht 33342 stain nuclear stain. Scale bar= 20μM.

Figure S5 The effect of acetate and propionate on the expression of L-cell transcription factors in NCI-H716 cells, related to Figure 6.

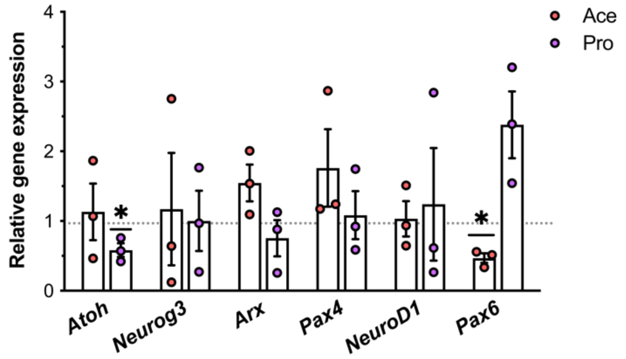

Relative gene expression of *Atoh1, Neurog3, Arx, Pax4, NeuroD1* and *Pax6)* in NCI-H716 cells treated for 24h with 2mM of sodium acetate (Ace) or sodium propionate (Pro). Transcript levels were detected by RT-qPCR and gene expression relative to untreated was determined using the $2^{-\Delta Ct}$ method with ribosomal protein L12 (*Rpl12)* as a housekeeping gene control. Data are presented as the mean ± SEM of data collected across three independent experiments in which conditions were run in at least triplicate. Symbols depict the mean ± SEM of individual biological repeats. (*p<0.05, One sample t-test; Untreated (1) vs SCFA).

Table S1 Resources used in this study, related to Methods

Key Resources Table

REAGENT or RESOURCE	SOURCE	IDENTIFIER
Antibodies		
Mouse monoclonal anti-GLP-1	Abcam	Cat# ab23468; RRID: AB_470325
Rabbit polyclonal anti-PYY	Abcam	Cat# ab22663; RRID: AB_2175186
Mouse monoclonal Anti-FLAG M1	Sigma-Aldrich	Cat# F3040; RRID:AB_439712
Chemicals, peptides, and recombinant proteins		
Advanced DMEM	Gibco	Cat# 12491015
AZ-1729	Tocris	Cat# 6826
B27	Life Technologies	Cat# 17504044
CHIR99021	Reprocell	Cat# 04-0004
Dyngo-4a	Abcam	Cat# ab120689
Forskolin	Abcam	Cat# ab120058
GLPG0974	Sigma-Aldrich	Cat# SML2443
N2	Life Technologies	Cat# 17502001
Cultrex Basement Membrane Extract	Bio-Techne	Cat# 3432-010-01
Matrigel	Corning	Cat# 356231
Pertussis toxin	Tocris	Cat# 3097
Recombinant Murine EGF	PeproTech	Cat# 315-09
Recombinant Murine Noggin	PeproTech	Cat# 315-09
Recombinant Murine R-Spondin	Sino Biological	Cat# 50316-M08S
RPMI-1640		Cat# R8785
Y-27632	Sigma-Aldrich	Cat# Y0503
YM-254890	Tocris	Cat# 7352
3-isobutyrl-1-methylxanthine (IBMX)	Sigma-Aldrich	Cat# I5879
Critical commercial assays		
Human PYY (Total) ELISA Kit	Merck	Cat# EZHPYYT66K
Fluo-4 Direct Calcium Assay	Invitrogen	Cat# F10471
HTRF cAMP Gs Detection kit	Revvity	
HTRF IP-One Gαq Detection Kit	Revvity	Cat# 62IPAPEB
Deposited data		
Raw single-cell RNA-seq (sc-RNASeq) data	Tabula Muris Consortium [39]	GEO: GSM2967048
Bulk RNA-seq data	This paper	GEO: GSE320030
Experimental models: Cell lines		
Human: NCI-H716 cells	ATCC	Cat# CCL251; RLID: CVCL_1581
Mouse: Hes1-GFP organoid	This paper	N/A
Experimental models: Organisms/strains		
Mouse: $Hes1^{GFP(-/+)}$	Sage Lab	[33]
Oligonucleotides		
Primers for RT-qpCR, see Table S1		
Forward primer for PCR cloning FLAG-hFFAR2: 5'- GCTACCTGGGAGTGGCTTTC	This paper	N/A
Reverse primer for PCR cloning FLAG-hFFAR2:	This paper	N/A

5'- ATAACCCAGGCCACCAGAG		
Recombinant DNA		
Human GPR43 ORF Clone	Fisher Scientific	Cat# 17307384; NM_005306
Plasmid: Human FLAG-FFAR2	This manuscript	N/A
Software and algorithms		
ImageJ2	Schneider et al [90]	V2.9.0/1.53t https://imagej.net/ij/
JaCoP ImageJ plugin	Bolte & Cordelieres [91]	https://imagej.net/plugins/jacop
R Studio	Posit, PBC	V2023.09.0+463 https://posit.co/download/rstudio-desktop/
Seurat	Satija et al [87]	V4 https://satijalab.org/seurat/articles/install.html
DESeq2	Love et al [92]	V1.48.2 https://bioconductor.org/packages/DESeq2/
Msigdbr	Liberzon et al [89]	V25.1.1 https://CRAN.R-project.org/package=msigdbr
Singscore	Foroutan et al [40]	V1.28.1 https://davislaboratory.github.io/singscore/

Table S2 List of primers used in this study, related to Methods

Primers for used for Real-Time Quantitative PCR (RT-qPCR)

Gene	Forward	Reverse
Ffar2	5'- GCTACCTGGGAGTGGCTTTC	5'- CATAACCCAGGCCACCAGAG
Ffar3	5'- CCAATGGGACCTGCTACCTG	5'- ATCAGCGGGACCACAAAGAG
Gcg	5'- TTCAGACCAAAATCACTGAC	5'- AACATTTCAAACATCCCACG
Pyy	5'- TCTTTTCCCATACCGCTGCC	5'- CTCGTCTGCTTCACAAGCTATC
Atoh1	5'- CCTTCCAGCAAACAGGTGAAT	5'- TTGTTGAACGACGGGATAACAT
Neurog3	5'- GCGCCGGTAGAAAGGATGA	5'- GGTCACTTCGTCTTCCGAGG
Arx	5'- TGGAAACAGAGGACCAGCAC	5'- GTTGGAGTTGGAGCGAGGTT
Pax4	5'- ATACCCGGCAGCAGATTGTG	5'- AAGACACCTGTGCGGTAGTAA
NeuroD1	5'- ATGACCAAATCGTACAGCGAG	5'- GTTCATGGCTTCGAGGTCGT
Pax6	5'- CAGACACAGCCCTCACAAACAC	5'- TGGTGAAGCTGGGCATAGG
Rpl12	5'- CATCTCCTTCTCGGCATCA	5'- AACCCTGTTGTCAATGCCTC
