# Supplementary material for: A selective and augmentable butyrate-FFAR2 signal circuitry programs the cellular identity of enteroendocrine L-cells
Source: Commun Biol. 2026 Mar 17;9:606. doi: 10.1038/s42003-026-09830-5 (PMC13144487; doi:10.1038/s42003-026-09830-5)
Supplement: Supplementary file 3 — Description of Additional Supplementary Files [file 42003_2026_9830_MOESM3_ESM.pdf]

Description of Additional Supplementary Files

File name: Supplementary Data
Description: Numerical Source Data.
