# Supplementary material for: A selective and augmentable butyrate-FFAR2 signal circuitry programs the cellular identity of enteroendocrine L-cells
Source: Commun Biol. 2026 Mar 17;9:606. doi: 10.1038/s42003-026-09830-5 (PMC13144487; doi:10.1038/s42003-026-09830-5)
Supplement: Supplementary file 5 — Reporting Summary [file 42003_2026_9830_MOESM5_ESM.pdf]

nature portfolio

Corresponding author(s): DAPR. COMMSBIO-25-7631A.

Last updated by author(s): Feb 6, 2025

Reporting Summary

Nature Portfolio wishes to improve the reproducibility of the work that we publish. This form provides structure for consistency and transparency in reporting. For further information on Nature Portfolio policies, see our Editorial Policies and the Editorial Policy Checklist.

Statistics

For all statistical analyses, confirm that the following items are present in the figure legend, table legend, main text, or Methods section.

n/a	Confirmed	
☐	☒	The exact sample size (*n*) for each experimental group/condition, given as a discrete number and unit of measurement
☐	☒	A statement on whether measurements were taken from distinct samples or whether the same sample was measured repeatedly
☐	☒	The statistical test(s) used AND whether they are one- or two-sided<br>*Only common tests should be described solely by name; describe more complex techniques in the Methods section.*
☐	☒	A description of all covariates tested
☐	☒	A description of any assumptions or corrections, such as tests of normality and adjustment for multiple comparisons
☐	☒	A full description of the statistical parameters including central tendency (e.g. means) or other basic estimates (e.g. regression coefficient) AND variation (e.g. standard deviation) or associated estimates of uncertainty (e.g. confidence intervals)
☐	☐	For null hypothesis testing, the test statistic (e.g. *F*, *t*, *r*) with confidence intervals, effect sizes, degrees of freedom and *P* value noted<br>*Give P values as exact values whenever suitable.*
☒	☐	For Bayesian analysis, information on the choice of priors and Markov chain Monte Carlo settings
☒	☐	For hierarchical and complex designs, identification of the appropriate level for tests and full reporting of outcomes
☒	☐	Estimates of effect sizes (e.g. Cohen's *d*, Pearson's *r*), indicating how they were calculated

Our web collection on statistics for biologists contains articles on many of the points above.

Software and code

Policy information about availability of computer code

Data collection	Confocal images were acquired using a Leica Stellaris 8 Inverted confocal microscope (Leica) with the LasX software. For capturing super-resolution confocal images, the LIGHTNING detection module on Leica Stellaris 8 was applied, utilising a pinhole size of 0.5 Airy Units (AU) and adaptive deconvolution. For widefield fluorescence microscopy of live organoid cultures, images were using Keyence All in One Fluorescence BZ-X Microscope and software.
Data analysis	Image analysis was carried out on ImageJ2. JACoP v2.1.3 plugin on ImageJ was used for colocalisation analysis.<br>All statistical data analyses were performed on GraphPad Prism.<br>Single-cell RNA-seq data from the processed using the Seurat package in R Studio.<br>Bulk RNA-seq analysis was processed using DESeq2 and the SingScore package in R Studio.

For manuscripts utilizing custom algorithms or software that are central to the research but not yet described in published literature, software must be made available to editors and reviewers. We strongly encourage code deposition in a community repository (e.g. GitHub). See the Nature Portfolio guidelines for submitting code & software for further information.

Data

Policy information about availability of data

All manuscripts must include a data availability statement. This statement should provide the following information, where applicable:

- Accession codes, unique identifiers, or web links for publicly available datasets
- A description of any restrictions on data availability
- For clinical datasets or third party data, please ensure that the statement adheres to our policy

Single-cell RNA-seq data from the Tabula Muris Project, generated by FACS-based full-length transcript analysis, were downloaded from the Gene Expression Omnibus (GEO; accession GSM2967048).
Bulk RNA-seq data, including Gene Counts, MetaData and output of SingScore analysis, are publicly available at https://doi.org/10.6084/m9.figshare.30925859.v1

Research involving human participants, their data, or biological material

Policy information about studies with human participants or human data. See also policy information about sex, gender (identity/presentation), and sexual orientation and race, ethnicity and racism.

Reporting on sex and gender	N/A
Reporting on race, ethnicity, or other socially relevant groupings	N/A
Population characteristics	N/A
Recruitment	N/A
Ethics oversight	N/A

Note that full information on the approval of the study protocol must also be provided in the manuscript.

Field-specific reporting

Please select the one below that is the best fit for your research. If you are not sure, read the appropriate sections before making your selection.

☒ Life sciences ☐ Behavioural & social sciences ☐ Ecological, evolutionary & environmental sciences

For a reference copy of the document with all sections, see nature.com/documents/nr-reporting-summary-flat.pdf

Life sciences study design

All studies must disclose on these points even when the disclosure is negative.

Sample size	No formal sample size calculation was performed prior to the study. Sample sizes were initially selected based on precedent from functional characterisation of receptor–ligand signalling in heterologous systems and similar intestinal cell lines (Le Poul et al, 2003; Brown et al, 2003; Bolognini et al, 2016; Caengprasath et al, 2020) where robust effects were observed.<br><br>For exploratory experiments without prior knowledge of expected effect sizes, n = 3–4 biological replicates per group were used in line with field standards to balance capturing biological variability with practical feasibility. These exploratory datasets informed the design and effect size estimates for subsequent experiments.
Data exclusions	Data from individual passages would have been excluded from all downstream analyses if they failed to meet pre-established quality control criteria. These criteria were: (i) cell viability, determined by Trypan Blue exclusion, <90%; (ii) in functional assays, positive/negative control responses or reference gene cycle threshold (Ct) values outside of three standard deviations from the mean values established during pilot assay validation. For NCI-H716 cells, functional assay controls included forskolin, IBMX, and lithium chloride for signalling assays; the RPL12 housekeeping gene for qPCR; and, for ICC/IF imaging, a primary-only antibody control. For HES1-GFP organoids, the functional assay control was the Notch inhibitor DBZ; (iii) supernatants testing positive for mycoplasma contamination. No data were excluded on the basis of these criteria.
Replication	All experiments were performed with three technical replicates per condition and repeated in ≥3 independent biological experiments across distinct cell passages except bulk RNA-seq which was carried out in single replicates in n=1 repeats. For NCI-H716 assays, independent experiments were conducted at passages 19, 24, 29, and 34. Intra-assay variability, calculated as the CV% between technical replicates within the same experiment, was consistently <20%. Pre-defined QC thresholds (cell viability ≥90%; control samples and reference gene Ct within ±3 SD of pilot-validation means; mycoplasma negative) were met in all independent biological runs.
Randomization	Samples and treatments were allocated systematically across culture vessels to reduce potential edge and batch effects. Experimental replicates were pooled and processed under standardized conditions to ensure consistency and reproducibility. Data were normalized within

each experimental repeat to appropriate internal positive controls for both untreated and treated groups, minimizing technical variability and enabling reliable comparison across replicates.

Blinding

Blinding was not feasible during experimental setup due to the nature of the cell culture treatments and the fact that all experiments were conducted by a single researcher. However, to minimize potential bias during data analysis, particularly for imaging-based assays, file names were coded to mask treatment identity during processing and quantification.

Reporting for specific materials, systems and methods

We require information from authors about some types of materials, experimental systems and methods used in many studies. Here, indicate whether each material, system or method listed is relevant to your study. If you are not sure if a list item applies to your research, read the appropriate section before selecting a response.

Materials & experimental systems

n/a	Involved in the study
☐	☒ Antibodies
☐	☒ Eukaryotic cell lines
☒	☐ Palaeontology and archaeology
☒	☐ Animals and other organisms
☒	☐ Clinical data
☒	☐ Dual use research of concern
☒	☐ Plants

Methods

n/a	Involved in the study
☒	☐ ChIP-seq
☒	☐ Flow cytometry
☒	☐ MRI-based neuroimaging

Antibodies

Antibodies used

Mouse monoclonal anti-GLP-1, Abcam, Cat# ab23468, Clone number 11E2
Rabbit polyclonal anti-PYY, Abcam, Cat# ab22663
Mouse monoclonal anti-FLAG M1, Sigma-Aldrich Cat# F3040, Clone number M1

Validation

All primary antibodies were validated for use in ICC/IF on manufacturer's websites.

Eukaryotic cell lines

Policy information about cell lines and Sex and Gender in Research

Cell line source(s)

NCI-H716 cell line: ATCC, CCL-251.

Authentication

Cell line was authenticated by ATCC.

Mycoplasma contamination

All cell lines were negative for Mycoplasma contamination.

Commonly misidentified lines
(See ICLAC register)

N/A

Plants

Seed stocks

N/A

Novel plant genotypes

N/A

Authentication

N/A
